# Correlations between ASCC3 Gene Polymorphisms and Chronic Hepatitis B in a Chinese Han Population

**DOI:** 10.1371/journal.pone.0141861

**Published:** 2015-11-04

**Authors:** Lifeng Liu, Jinliang Zhang, Yan Lu, Chunfang Fang, Senlin Li, Jusheng Lin

**Affiliations:** 1 Department of Gastroenterology, Liaocheng People’s Hospital, Liaocheng Clinical School of Taishan Medical University, Liaocheng, Shandong, China; 2 Department of Hepatobiliary Surgery, Liaocheng People’s Hospital, Liaocheng Clinical School of Taishan Medical University, Liaocheng, Shandong, China; 3 Institute of Liver Disease, Tongji Hospital, Tongji Medical College, Huazhong University of Science and Technology, Wuhan, Hubei, China; University of Pisa, ITALY

## Abstract

We have previously identified 8 SNPs in Han Chinese HBV carriers that are associated with disease progression. Although not well studied, genetic factors may also play a significant role in developing chronic HBV disease after exposure. We extend the effect of these eight SNPs on persistent HBV infection in this study. A total of 875 unrelated Han Chinese, 493 chronic hepatitis B subjects (CHB) and 382 HBV clearance individuals (Clear), were recruited from Hubei Province from September 2007 to March 2010. SNPs were verified by using TaqMan 7900HT Sequence Detection System. By using multiple logistic regression analysis, each of the 8 SNP associations was tested using 3 different genetic models (Dominant, Recessive and Additive model), in 4 types of analyses (full sample, men, women, age stratified). A Bonferroni correction was used to account for multiple statistical tests for each SNP association (P<0.05/8 = 0.0063). A significant correlation was observed at SNP rs10485138 located in ASCC3 gene in female patients (OR, 0.445; 95% CI, 0.253–0.784; *P* = 0.005). Females bearing C allele infected by HBV had an increased susceptibility to CHB compared with those T allele carriers. Our results indicated that SNP rs10485138 located in ASCC3 gene was associated with persistent HBV infection in Han Chinese.

## Introduction

Hepatitis B virus (HBV) infection is a global health issue, with approximately2 billion people exposed to HBV and 350 million of them suffering from persistent HBV infection [1]. The prevalence of HBV infection vary greatly worldwide, with a relative low-incidence in western countries[2,3] and endemic in Asian and most of Africa countries. The incidence rate of HBV infection in China is 7.18% [4], causing high mortality and societal burden. The clinical outcome of HBV infection varies from spontaneous recovery to persistent infection. Approximately 80–90% of infants who infected HBV from their mothers would develop chronic hepatitis B. While more than 95% of HBV infection acquired in adulthood is resolved within 6 months with or without clinical symptoms, and less than 5% develop persistent infection[5]. HBV is not directly cause hepatic injury. After HBV infection, hepatic injury is thought to be due to immune responses of the host, which depends on an intricate interplay of host factors (such as age, gender, immune status), viral and environmental factors. The mechanisms underlying the different clinical outcomes of HBV infection are not fully understood. Researches about twins, family-clustering ofHBV infection and ethical studies of HBV infection indicate that host genetic background play an important role in HBV infection [6–9]. Host genetic factors, especially single nucleotide polymorphisms (SNPs), is regarded to be one of the determinants for this clinical heterogeneity. A number of polymorphisms in candidate genes, For example CXCL10[10], CD24[11], IFNAR1[12] Furin[13] have been validated for associations with HBV persistence. A Genome wide association studies (GWAS) using large samples identified that HLA-DPA1 and DPB1 were associated with persistent HBV infection in Japanese population [14].

Eight SNPs were previously identified to be associated with HBV progression in a Genome-Wide Association Study (GWAS) with DNA pooling carried out by ourself in 2011 (GSE26034). Four pools and twelve chips (each pool was replicated in triplicate) were performed using Affymetrix Genome-Wide Human Mapping SNP6.0 Arrays. This study included four groups: case A was acute liver failure group, case B was liver cirrhosis group, case C was hepatocellular carcinoma group\, and control was asymptomatic carrier group. One group corresponds to one pool. Raw probe intensity data (CEL files) were used to calculate relative allele signal (RAS) scores and to estimate allele frequencies. The silhouette method was used to analyze the results of DNA pooling. Eight SNPs (rs11866328, rs10845858, rs1041236, rs2013562, rs786100, rs12206945, rs10485138, rs6909880) that were top predictors in the DNA pooling screening stage (Silhouette method exceeding 0.8) were selected. Rs11866328 is an intronic SNP in GRIN2A gene at chromosome 16. GRIN2A gene is associated with Parkinson disease [15] and speech dysfunction[16]. Rs10845858 is an intronic SNP in GRIN2B gene at chromosome 12. GRIN2B gene is associated with schizophrenia[17]. Rs1041236 is an intronic SNP in GPA33 gene at chromosome 1. GPA33 gene is expressed primarily in the normal intestine and in >95% of colon tumors but not by other normal tissues [18–20], and is a therapeutic target in colon cancer immunotherapy [21,22]. Rs2013562 is an intronic SNP in UGT2B4 gene at chromosome 4, a gene is associated with breast cancer [23]. Rs786100 is an intronic SNP in BNC2 gene at chromosome 9, BNC2 gene is associated with ovarian cancer[24]. Rs12206945, rs10485138 and rs6909880 are intronic SNPs in ASCC3 gene at chromosome 6. ASCC3 gene is associated with DNA repair and cell proliferation [25]. They were evaluated by using 854 asymptomatic HBV carriers (AsC) versus 1944 progressed HBV carriers (including liver cirrhosis, hepatocellular carcinoma and acute liver failure) in the Han Chinese population [26]. We performed a PubMed search and had not found a relevant report about these eight SNPs and its corresponding six genes with HBV persistence. In this study, we sought to extend correlations between these 8 SNPs and CHB in Han Chinese population.

## Patients and Methods

### Ethic statement

The study was approved by the local research ethics committee (REC) at the Tongji Hospital of Huazhong University of Science and Technology (China) in accordance with the principle of the Helsinki Declaration II. All written informed consent documents from each participant were obtained during the enrollment phase.

### Patients

A total of 875 unrelated Han Chinese were recruited from Hubei Province (Wuhan Tongji Hospital, Wuhan Union Hospital and Traditional Chinese Medicine Hospital of Hubei Province) from September 2007 to March 2010. A uniform questionnaire was used at enrollment including gender, age, self-report of HBV transmission, family history of HBV infection, alcohol consumption et al. The demographic information included birth-date, birthplace, past and current residency. This case-control study was composed of two subgroups: 382 HBV patients who cleared their infection spontaneously (Clear) and 493 chronic hepatitis B patients (CHB). The recruitment criteria were listed in S1 Table. Clear patients were at least 35 years old at enrollment to avoid potential confounding by HBV vaccination that became available since 1981; infant vaccination was introduced in 1992[27].

### DNA Isolation and SNP Genotyping

Genomic DNA was isolated from peripheral whole blood using TIANamp blood DNA kit (Tiangen Biotech [Beijing] Co., Ltd., China). The concentration and purity of the DNA were determined with a NanoDrop spectrophotometer and diluted to a final concentration of 8 ng/μL. Characteristics of 8 SNPs were shown in Table 1. The genotyping of genetic polymorphisms was performed via the TaqMan method according to the protocol of TaqMan SNP Genotyping Assays (AppliedBiosystems, California, USA). The TaqMan 7900HT Sequence Detection System (Applied Biosystems, Foster City, CA) was used for genotyping according to the manufacturer’s instructions. Each assay was carried out in a 5-μL reaction system consisting of TaqMan universal polymerase chain reaction master mix (2.5μL, Applied Biosystems), SNP genotyping assay (20X 0.25μL), genomic DNA (1.25μL 8ng/μL) and DNase-free, sterile-filtered water (1.0μL) in MicroAmp Optical 384-Well Reaction Plate with Barcode (4309849 Applied Biosystems, California, USA). The SNP genotyping assay included forward and reverse primers, and 6-carboxyfluorescein (FAM)- and 4,7,2¢-trichloro-7¢-phenyl-6-carboxyfluorescein (VIC)-labeled probes (ABI Assay on Demand, S2 Table). The program was heating to 95°C for 10 minutes followed by 45 cycles of 92°C for 15 seconds and 60°C for 1 minute. Allelic category was measured automatically using the Sequence Detection System 2.3 software (Applied Biosystems) according to the intensity of VIC and FAM dye. To validate the TaqMan assay, we analyzed 43 (5%) randomly selected samples by both direct sequencing (Invitrogen, Shanghai, China) and Taq-Man assay. The concordance rate of these two methods was 100%, indicating that the TaqMan assay was reliable.

**Table 1:** Characteristics of variants.

Affymetrix SNP ID	SNP	Chr	Location	Gene	Allele
SNP_A-1956120	rs11866328	16	9770057	GRIN2A	T/G
SNP_A-2031932	rs10845858	12	1404578	GRIN2B	A/G
SNP_A-1970901	rs1041236	1	165321604	GPA33	C/T
SNP_A-2084283	rs2013562	4	70389164	UGT2B4	T/C
SNP_A-4213388	rs786100	9	16794082	BNC2	A/G
SNP_A-8389359	rs12206945	6	101124753	ASCC3	G/A
SNP_A-4265842	rs10485138	6	101245311	ASCC3	C/T
SNP_A-1987038	rs6909880	6	101286075	ASCC3	G/T

SNP: single nucleotide polymorphisms, Chr: chromosome, Location: Genomic position (NCBI Build 36), Allele: minor allele/major allele, Clear: spontaneously recovered individuals with history of HBV infection, CHB: chronic hepatitis B patients.

### Statistical analysis

Statistical analysis was performed by using SPSS 17.0 and HaploView 4.2 software. Hardy-Weinberg equilibrium of genotype frequencies was evaluated online using the website of Technical University Munich (http://ihg2.helmholtz-muenchen.de/cgi-bin/hw/hwa1.pl). χ^2^ tests and independent sample t-test were used to examine the differences in clinical characteristics of participants. Tests were two-sided with a significance level of P < 0.05. The adjusted Odds ratio (OR) and 95% confidence interval (95% CI) were calculated by multiple logistic regression under genetic models with adjustment for gender and age. The linkage disequilibrium (LD) was performed using the HaploView 4.2 softwareA Bonferroni correction was used to account for multiple statistical tests for each SNP association. P<0.05/8 = 0.0063 was the appropriate significance level

## Results

### Hardy-Weinberg equilibrium test

Significant difference was not found between observed and expected frequencies of each genotype in involved populations (P ≥0.05 Results were listed in S3 Table). This indicated that these participants had a relatively stable genetic background and were suitable for further genetic statistical analysis.

### Clinic and demographic characteristics

The clinical and demographic characteristics of this study were summarized in Table 2, including gender, age, drinkers, family history of HBV infection, serum markers of hepatitis B virus, serum level of HBV-DNA load, alanine transaminase (ALT) and aspartate aminotransferase (AST). “Drinker” was defined as alcohol consumption of >40 g/wk for men and >20 g/wk for women [10]. Although an effort was made to obtain a good match on gender and age between Clear and CHB, there were more men in CHB (P < 0.001) and subjects in CHB were much younger (P < 0.001) in our hospital-based case-control study. There was no significant difference in alcohol consumption between Clear and CHB. All Clears were 35 years or older, while the number was 314 in CHB, and 179 CHB under 35 years. Nity-fiveCHB patients were HBeAg positive and the percentage was 19.27%. Seventy CHB patients had family history of HBV infection and the percentage was 14.2%. There was a significant difference in serum ALT and AST level (P < 0.001) between Clear and CHB.

**Table 2:** Clinical and demographic characteristics of participants.

	Clear (n = 382)	CHB (n = 493)	P value
Gender (M/F)	174/208	408/85	P < 0.001
Age (Y), (n, mean±SD)			
≥35y	382 (49.45 ±8.70)	314 (44.05±7.94)	P< 0.001
<35	-	179 (27.35±4.68)	
Drinkers, n (%)	98 (25.65)	136 (27.31)	P = 0.261
HBsAg	All -	All +	
Anti-HBs IgG	All +	All -	
HBeAg, n (%)	All -	95 (19.27)	
Anti-HBc IgG	All +	All +	
Family history no. (%)	No.	70 (14.20)	
ALT (IU/L)	32.16 (3.25)	189.02 (328.76)	P < 0.001
AST (IU/L)	25.78 (7.90)	163.52 (287.27)	P < 0.001
HBV-DNA (copy/ml)	All -	4.23 (1.61)	

M: male, F: female, Y: years, SD: standard deviation, no.: number, Clear: spontaneously recovered individuals with history of HBV infection, CHB: chronic hepatitis B patients. ALT: alanine transaminase, AST: aspartate aminotransferase, No means non-detected. The differences of clinical characteristics were calculated using chi-square test and independent sample t-test.

### Logistic regression analysis of the polymorphisms

Multiple logistic regressions with adjustment for sex and age was used to determine SNPs’ effects on CHB in comparison with Clear. Each of the 8 SNP associations was tested using 3 different genetic models (Dominant model, Recessive model and Additive model), in 4 types of analyses (full sample, men only, women only, age stratified). Genotype-based additive model and recessive model, and an allele-based model were shown. The best-fit effect of these eight SNPs was observed in the allele model in women only.

**Fig 1:**
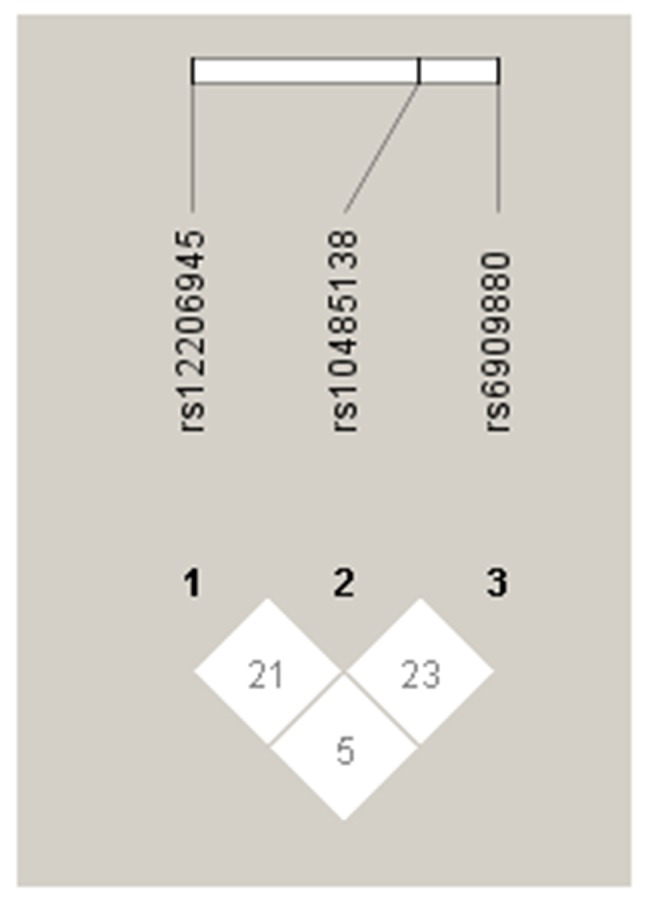
Linkage disequilibrium analysis of the SNPs rs12206945, rs10485138, and rs6909880 located in ASCC3 gene in HBV clearance population (n = 382) generated by HaploView 4.2 software.

Associations in full sample were listed in Table 3, and no differences of variants in genotype distributions or allele frequencies were detected. Analysis in men only was summarized in Table 4 and associations in genotype or allele distributions were not found. Correlation in women was displayed in Table 5. A significant correlation was observed at rs10485138 located in ASCC3 gene in allele frequencies. Females bearing C allele infected by HBV had an increased susceptibility to CHB compared with those T allele carriers (OR, 0.445; 95% CI, 0.253–0.784; *P* = 0.005). A slight difference was observed at SNP rs1041236 located in GPA33 gene in genotype distribution at p <0.05. Females carrying TT homozygote had higher susceptible to CHB compared with CC carriers under the additive model (OR, 0.099; 95% CI, 0.010–0.968; *P* = 0.047). The significance do not survived after Bonferroni correction. To decrease age distinction, an analysis for patients equal or over 35 years was performed, and no significant difference was found (Table 6). Three SNPs (rs12206945, rs10485138 and rs10485138) were all located in ASCC3 gene in Chr 6. Though these three SNPs had no significant associations with CHB, we further constructed the linkage disequilibrium (LD) using HaploView 4.2 software using frequencies obtained from the Clear group (n = 382). Strong LD was not detected between these three SNPs (D’ < 0.25, and r^2^<0.01 Fig 1). We further calculated association between SNP rs10485138 and HBeAg status in CHB group. Logistic regression under 3 different genetic models (Dominant model, Recessive model and Additive model) was used. Compared with HBeAg negative patients, no significance was found (results were listed in Table 7).

**Table 3:** Association results for SNPs between Clear and CHB patients.

Polymorphisms	Allele	Clear (n = 382)	CHB (n = 493)	Clear vs.CHB	
				P	OR (95%CI)
rs11866328	TT	22 (0.06)	17 (0.04)		
	TG	123 (0.32)	159 (0.32)	0.482	1.451 (0.515–4.088)^a^
	GG	237 (0.62)	317 (0.64)	0.416	1.441 (0.598–3.476) ^b^
	GG+TG	360 (0.94)	476 (0.96)	0.376	1.483 (0.619–3.552) ^c^
	T	167 (0.22)	193 (0.20)		
	G	597 (0.78)	793 (0.80)	0.404	1.139 (0.839–1.548) ^d^
rs10845858	AA	59 (0.15)	87 (0.18)		
	AG	205 (0.54)	254 (0.51)	0.399	0.800 (0.476–1.344) ^a^
	GG	118 (0.31)	152 (0.31)	0.537	0.837 (0.477–1.471) ^b^
	AG+GG	323 (0.85)	406 (0.82)	0.406	0.817 (0.507–1.316)^c^
	A	323 (0.42)	428 (0.43)		
	G	441 (0.58)	558 (0.57)	0.736	0.958 (0.747–1.229)^d^
rs1041236	TT	208 (0.55)	253 (0.51)		
	TC	154 (0.40)	198 (0.40)	0.381	0.857 (0.608–1.210) ^a^
	CC	20 (0.05)	42 (0.09)	0.785	1.108 (0.528–2.325) ^b^
	TC+CC	174 (0.45)	250 (0.49)	0.326	0.837 (0.587–1.193)^c^
	T	570 (0.75)	704 (0.71)		
	C	194 (0.25)	282 (0.29)	0.490	0.905 (0.683–1.200) ^d^
rs2013562	CC	81 (0.21)	98 (0.20)		
	CT	187 (0.49)	246 (0.50)	0.150	1.421 (0.881–2.293) ^a^
	TT	114 (0.30)	149 (0.30)	0.286	1.393 (0.758–2.560) ^b^
	CT+TT	301 (0.79)	395 (0.80)	0.158	1.392 (0.880–2.202) ^c^
	C	349 (0.46)	442 (0.45)		
	T	415 (0.54)	544 (0.55)	0.654	1.059 (0.824–1.360)^d^
rs7861010	AA	30 (0.08)	32 (0.06)		
	AG	148 (0.39)	203 (0.41)	0.906	1.046 (0.497–2.201) ^a^
	GG	204 (0.53)	258 (0.53)	0.579	0.813 (0.392–1.688)^b^
	AG+GG	352 (0.92)	461 (0.94)	0.719	0.885 (0.457–1.716) ^c^
	A	208 (0.27)	267 (0.27)		
	G	556 (0.73)	719 (0.73)	0.188	0.826 (0.621–1.098) ^d^
rs12206945	GG	26 (0.07)	38 (0.08)		
	GA	144 (0.38)	185 (0.38)	0.894	1.055 (0.482–2.308) ^a^
	AA	212 (0.55)	270 (0.54)	0.777	0.894 (0.410–1.947) ^b^
	GA+AA	356 (0.93)	455 (0.92)	0.925	0.966 (0.468–1.994) ^c^
	G	264 (0.35)	261 (0.26)		
	A	500 (0.65)	725 (0.74)	0.321	0.863 (0.644–1.155) ^d^
rs10485138	TT	15 (0.04)	14 (0.03)		
	TC	120 (0.31)	155 (0.31)	0.669	1.285 (0.407–4.058) ^a^
	CC	247 (0.65)	324 (0.66)	0.896	1.077 (0.352–3.291)^b^
	TC+CC	367 (0.96)	479 (0.97)	0.779	1.164 (0.404–3.351) ^c^
	T	150 (0.20)	183 (0.19)		
	C	614 (0.80)	803 (0.81)	0.255	0.826 (0.595–1.148) ^d^
rs6909880	GG	38 (0.10)	32 (0.06)		
	GT	153 (0.40)	186 (0.38)	0.303	1.456 (0.712–2.975) ^a^
	TT	191 (0.50)	275 (0.56)	0.837	0.926 (0.446–1.924) ^b^
	GT+TT	344 (0.90)	461 (0.94)	0.791	1.092 (0.569–2.099) ^c^
	G	229 (0.30)	250 (0.25)		
	T	535 (0.70)	736 (0.75)	0.817	0.968 (0.738–1.271)^d^

SNP: single nucleotide polymorphisms, Clear: spontaneously recovered individuals with history of HBV infection, CHB: chronic hepatitis B patients. P values, ORs and 95% CIs were calculated by multiple logistic regression adjusting for gender and age; - means differences could not be detected.

^a^ means additive model: the first genotype/the second genotype

^b^means additive model: the first genotype/the third genotype

^c^means recessive model: the first genotype/the second +the third genotype

^d^means allele model.

**Table 4:** Stratification analysis for sex between Clear and CHB in male patients.

Polymorphisms	Allele	Clear (n = 174)	CHB (n = 408)	Clear vs.CHB	
				P	OR (95%CI)
rs11866328	TT	11 (0.06)	11 (0.03)		
	TG	56 (0.32)	132 (0.32)	0.236	2.020 (0.591–6.907) ^a^
	GG	107 (0.62)	265 (0.65)	0.278	1.838 (0.612–5.515)^b^
	GG+TG	163 (0.94)	397 (0.97)	0.247	1.878 (0.646–5.640) ^c^
	T	78 (0.22)	154 (0.19)		
	G	270 (0.78)	662 (0.81)	0.268	1.231(0.852–1.781) ^d^
rs10845858	AA	31 (0.18)	72 (0.18)		
	AG	91(0.52)	214 (0.52)	0.888	1.047 (0.553–1.983) ^a^
	GG	52 (0.30)	122 (0.30)	0.897	0.955 (0.474–1.924) ^b^
	AG+GG	143 (0.82)	336 (0.82)	0.988	1.005 (0.562–1.796)^c^
	A	153 (0.44)	358 (0.44)		
	G	195 (0.56)	458 (0.56)	0.933	0.987 (0.731–1.334) ^d^
rs1041236	TT	96 (0.55)	202 (0.49)		
	TC	64 (0.37)	170 (0.42)	0.918	1.024 (0.649–1.618) ^a^
	CC	14 (0.08)	36 (0.09)	0.879	0.937 (0.405–2.168) ^b^
	TC+CC	78 (0.45)	206 (0.51)	0.948	1.014 (0.661–1.557) ^c^
	T	256 (0.74)	574 (0.70)		
	C	92 (0.26)	242 (0.30)	0.952	0.990 (0.699–1.385) ^d^
rs2013562	CC	43 (0.25)	81 (0.20)		
	CT	86 (0.49)	197 (0.48)	0.171	1.492 (0.842–2.647) ^a^
	TT	45 (0.26)	130 (0.32)	0.14	1.705 (0.839–3.465) ^b^
	CT+TT	131 (0.75)	327 (0.80)	0.145	1.487 (0.872–2.536)^c^
	C	172 (0.49)	359 (0.44)		
	T	176 (0.51)	457 (0.56)	0.211	1.214 (0.896–1.645) ^d^
rs7861010	AA	10 (0.06)	31 (0.08)		
	AG	66 (0.38)	148 (0.36)	0.083	0.421 (0.158–1.120) ^a^
	GG	98 (0.56)	229 (0.56)	0.111	0.486 (0.200–1.181)^b^
	AG+GG	164 (0.94)	377 (0.92)	0.078	0.467 (0.200–1.089) ^c^
	A	86 (0.25)	210 (0.26)		
	G	262 (0.75)	606 (0.74)	0.996	- ^d^
rs12206945	GG	10 (0.06)	34 (0.08)		
	GA	61 (0.35)	131 (0.32)	0.32	0.591 (0.210–1.665) ^a^
	AA	103 (0.59)	243 (0.60)	0.492	0.713 (0.272–1.870) ^b^
	GA+AA	164 (0.94)	374 (0.92)	0.383	0.663 (0.263–1.669) ^c^
	G	81 (0.23)	199 (0.24)		
	A	267 (0.77)	616 (0.76)	0.389	0.852 (0.593–1.226) ^d^
rs10485138	TT	7 (0.04)	11 (0.03)		
	TC	46 (0.26)	108 (0.26)	0.943	1.054 (0.246–4.513) ^a^
	CC	121 (0.70)	289 (0.71)	0.814	1.165 (0.327–4.151)^b^
	TC+CC	167 (0.96)	397 (0.49)	0.832	1.140 (0.339–3.837) ^c^
	T	60 (0.17)	130 (0.16)		
	C	288 (0.83)	686 (0.84)	0.717	1.078 (0.718–1.620)^d^
rs6909880	GG	17 (0.10)	26 (0.06)		
	GT	71 (0.41)	147 (0.36)	0.253	1.726 (0.677–4.405) ^a^
	TT	86 (0.49)	235 (0.58)	0.988	1.007 (0.405–2.504) ^b^
	GT+TT	157 (0.90)	382 (0.47)	0.627	1.229 (0.536–2.818) ^c^
	G	105 (0.30)	199 (0.24)		
	T	243 (0.70)	617 (0.76)	0.564	1.103 (0.791–1.537) ^d^

SNP: single nucleotide polymorphisms, Clear: spontaneously recovered individuals with history of HBV infection, CHB: chronic hepatitis B patients. P values, ORs and 95% CIs were calculated by multiple logistic regression adjusting for age; - means differences could not be detected.

^a^ means additive model: the first genotype/the second genotype

^b^means additive model: the first genotype/the third genotype

^c^means recessive model: the first genotype/the second +the third genotype

^d^means allele model.

**Table 5:** Stratification analysis for sex between Clear and CHB in female patients.

Polymorphisms	Allele	Clear (n = 208)	CHB (n = 85)	Clear vs.CHB	
				P	OR (95%CI)
rs11866328	TT	11 (0.05)	6 (0.07)		
	TG	67 (0.32)	27 (0.32)	0.911	1.166 (0.080–17.071) ^a^
	GG	130 (0.63)	52 (0.61)	0.334	0.441 (0.084–2.321) ^b^
	TG+GG	197 (0.95)	79 (0.93)	0.410	0.502 (0.097–2.590)^c^
	T	89 (0.21)	39 (0.23)		
	G	327 (0.79)	131 (0.77)	0.468	0.807 (0.453–1.439)^d^
rs10845858	AA	27 (0.13)	15 (0.18)		
	AG	115 (0.55)	40 (0.47)	0.108	0.430 (0.153–1.204) ^a^
	GG	66 (0.32)	30 (0.35)	0.486	0.648 (0.191–2.195) ^b^
	AG+GG	181 (0.44)	70 (0.41)	0.115	0.477 (0.190–1.198) ^c^
	A	169 (0.41)	70 (0.41)		
	G	247 (0.59)	100 (0.59)	0.777	0.932 (0.573–1.516) ^d^
rs1041236	TT	112 (0.54)	51 (0.60)			
	TC	90 (0.43)	28 (0.33)	0.409	0.639 (0.221–1.849) ^a^
	CC	6 (0.03)	6 (0.07)	0.047	0.099 (0.0010–0.968) ^b^
	TC+CC	96 (0.46)	34 (0.40)	0.978	0.987 (0.380–2.562) ^c^
	T	314 (0.75)	130 (0.76)		
	C	102 (0.25)	40 (0.24)	0.492	1.304 (0.613–2.770) ^d^
rs2013562	CC	38 (0.18)	17 (0.2)			
	CT	101 (0.49)	49 (0.58)	0.363	1.594 (0.583–4.357) ^a^
	TT	69 (0.33)	19 (0.22)	0.991	0.990 (0.173–5.673) ^b^
	CT+TT	170 (0.82)	68 (0.4)	0.597	1.308 (0.483–3.544) ^c^
	C	177 (0.43)	83 (0.49)		
	T	239 (0.57)	87 (0.51)	0.421	0.819 (0.502–1.334) ^d^
rs7861010	AA	20 (0.10)	1 (0.01)		
	AG	82 (0.39)	55 (0.65)	0.994	- ^a^	
	GG	106 (0.51)	29 (0.34)	0.992	- ^b^	
	AG+GG	188 (0.90)	84 (0.99)	0.994	- ^c^	
	A	122 (0.29)	57 (0.34)		
	G	294 (0.71)	11 (0.66)	0.836	1.078 (0.530–2.192) ^d^
rs12206945	GG	16 (0.08)	4 (0.05)		
	GA	83 (0.40)	54 (0.63)	0.382	2.185 (0.379–12.584) ^a^
	AA	109 (0.52)	27 (0.32)	0.620	1.624 (0.239–11.051) ^b^
	GA+AA	192 (0.92)	81 (0.95)	0.502	1.773 (0.334–9.418) ^c^
	G	115 (0.28)	62 (0.36)		
	A	301 (0.72)	108 (0.64)	0.349	0.773 (0.451–1.324)^d^
rs10485138	TT	8 (0.04)	3 (0.04)		
	TC	74 (0.36)	47 (0.55)	0.385	0.232 (0.009–6.243) ^a^
	CC	126 (0.60)	35 (0.41)	0.187	0.096 (0.003–3.110) ^b^
	TC+CC	200 (0.96)	82 (0.96)	0.239	0.161 (0.008–3.369) ^c^
	T	90 (0.22)	53 (0.31)		
	C	326 (0.78)	117 (0.69)	0.005	0.445 (0.253–0.784) ^d^
rs6909880	GG	21 (0.10)	6 (0.07)		
	GT	81 (0.39)	39 (0.46)	0.804	1.177 (0.326–4.246) ^a^
	TT	106 (0.51)	40 (0.47)	0.636	0.720 (0.185–2.808) ^b^
	GT+TT	187 (0.90)	79 (0.93)	0.838	0.888 (0.286–2.755) ^c^
	G	123 (0.30)	51 (0.30)		
	T	293 (0.70)	119 (0.70)	0.220	0.728 (0.439–1.209) ^d^

SNP: single nucleotide polymorphisms, Clear: spontaneously recovered individuals with history of HBV infection, CHB: chronic hepatitis B patients. P values, ORs and 95% CIs were calculated by multiple logistic regression adjusting for age; - means differences could not be detected.

^a^ means additive model: the first genotype/the second genotype

^b^means additive model: the first genotype/the third genotype

^c^means recessive model: the first genotype/the second +the third genotype

^d^means allele model.

**Table 6:** Stratification analysis for age ≥ 35 years between Clear and CHB.

Polymorphisms	Allele	Clear (n = 382)	CHB (n = 314)	Clear vs.CHB	
				P	OR (95%CI)
rs11866328	TT	22 (0.06)	10 (0.03)		
	TG	123 (0.32)	96 (0.31)	0.291	1.278 (0.677–3.681) ^a^
	GG	237 (0.62)	208 (0.66)	0.181	1.765 (0.767–4.056) ^b^
	GG+TG	360 (0.94)	305 (0.97)	0.205	1.702 (0.748–3.873) ^c^
	T	167 (0.22)	116 (0.18)		
	G	597 (0.78)	514 (0.82)	0.183	1.214 (0.912–1.615) ^d^
rs10845858	AA	58 (0.15)	54 (0.17)		
	AG	206 (0.54)	169 (0.54)	0.601	0.885 (0.559–1.400) ^a^
	GG	118 (0.31)	91 (0.29)	0.539	0.858 (0.526–1.399) ^b^
	AG+GG	324 (0.85)	260 (0.83)	0.557	0.877 (0.565–1.359) ^c^
	A	322 (0.42)	277 (0.44)		
	G	442 (0.58)	351 (0.56)	0.644	0.947 (0.752–1.192) ^d^
rs1041236	TT	208 (0.55)	177 (0.56)		
	TC	154 (0.40)	113 (0.36)	0.255	0.820 (0.583–1.154) ^a^
	CC	20 (0.05)	24 (0.08)	0.768	1.103 (0.573–2.123) ^b^
	TC+CC	174 (0.45)	137 (0.44)	0.331	0.851 (0.615–1.178) ^c^
	T	570 (0.75)	467 (0.74)		
	C	194 (0.25)	161 (0.26)	0.101	0.635 (0.369–1.092) ^d^
rs2013562	CC	81 (0.21)	55 (0.17)		
	CT	187 (0.49)	172 (0.55)	0.13	1.989 (0.817–4.842) ^a^
	TT	114 (0.30)	87 (0.28)	0.744	0.843 (0.301–2.356) ^b^
	CT+TT	301 (0.79)	259 (0.41)	0.342	1.524 (0.639–3.630) ^c^
	C	349 (0.46)	282 (0.45)		
	T	415 (0.54)	346 (0.55)	0.446	0.850 (0.559–1.292) ^d^
rs7861010	AA	30 (0.08)	26 (0.08)		
	AG	148 (0.39)	129 (0.41)	0.999	- ^a^
	GG	204 (0.53)	159 (0.51)	0.998	-^b^
	AG+GG	352 (0.92)	288 (0.92)	0.999	-^c^
	A	208 (0.27)	181 (0.29)		
	G	556 (0.73)	447 (0.71)	0.933	1.021 (0.623–1.674) ^d^
rs12206945	GG	26 (0.07)	24 (0.08)		
	GA	212 (0.55)	121 (0.38)	0.219	2.620 (0.565–12.160) ^a^
	AA	144 (0.38)	169 (0.54)	0.788	1.239 (0.261–5.879) ^b^
	GA+AA	356 (0.93)	290 (0.46)	0.837	1.171 (0.259–5.286) ^c^
	G	264 (0.35)	169 (0.27)		
	A	500 (0.65)	459 (0.73)	0.223	0.746 (0.466–1.195) ^d^
rs10485138	TT	15 (0.04)	9 (0.03)		
	TC	120 (0.31)	97 (0.31)	0.963	1.027 (0.334–3.163) ^a^
	CC	247 (0.65)	208 (0.66)	0.859	1.103 (0.373–3.260) ^b^
	TC+CC	367 (0.96)	305 (0.47)	0.885	1.082 (0.369–3.177) ^c^
	T	150 (0.20)	115 (0.18)		
	C	614 (0.80)	513 (0.82)	0.659	1.087 (0.751–1.572) ^d^
rs6909880	GG	38 (0.10)	22 (0.07)		
	GT	153 (0.40)	134 (0.43)	0.322	1.465 (0.688–3.119) ^a^
	TT	191 (0.50)	158 (0.50)	0.212	1.605 (0.763–3.375) ^b^
	GT+TT	344 (0.90)	292 (0.93)	0.24	1.541 (0.749–3.171) ^c^
	G	229 (0.30)	178 (0.28)		
	T	535 (0.70)	450 (0.72)	0.245	1.196 (0.884–1.617) ^d^

SNP: single nucleotide polymorphisms, Clear: spontaneously recovered individuals with history of HBV infection, CHB: chronic hepatitis B patients. P values, ORs and 95% CIs were calculated by multiple logistic regression adjusting for gender; - means differences could not be detected.

^a^ means additive model: the first genotype/the second genotype

^b^means additive model: the first genotype/the third genotype

^c^means recessive model: the first genotype/the second +the third genotype

^d^means allele model.

**Table 7:** Association of SNP rs10485138 with HBeAg status inCHB group.

genotype	HBeAg positive N = 95	HBeAg negative N = 398	P	OR 95% CI
TT	5	9	0.06	0.312 (0.094–1.036)^a^
TC	26	129	0.15	0.317 (0.066–1.528)^b^
CC	64	260	0.07	0.272 (0.067–1.109)^c^
			0.59	1.16 (0.673–1.999)^d^

^a^additive model TT/TC

^b^additive model TT/CC

^c^recessive model TT/TT+TC

^d^dominant model TT+TC/CC. Multiple logistic regression under three genetic models with adjustment for gender and age was used to test P value, OR and 95%CI.

## Discussion

In this study, we performed a hospital-based case-control study to find susceptible SNPs to HBV persistence in Han Chinese population, including 382 Clears and 493 CHB. Eight SNPs, which were top predictors of HBV progression in our previous GWAS, were genotyped by using TaqMan method. SNP rs10485138 located in ASCC3 gene had significant associations with CHB only in female patients. It is the first report about correlation between ASCC3 gene and persistent HBV infection.

Xu et al[28] examined Han Chinese population substructures in a diverse set of over 1700 Han Chinese samples collected from 26 administrative regions of China. Results showed that Han Chinese population is intricately substructured, with the main observed clusters corresponding roughly to northern Han (northern of Yangtze River), central Han (Shanghai, Jiangsu and Anhui province), and southern Han (southern of Yangtze River). All our participants were inquired birthplace, past and current residency at enrolment. They were all born from Hubei province (southern of Yangtze River), and were permanent resident. So we conclude that participants had relatively stable genetic backgrounds.

It is well documented that men are more easily to be infected with HBV than women [29]. Prevalence of HBsAg is significantly higher for males (8.6%) than females (5.7%) in China [27]. Reasons for gender discrepancy are complex. Some reports displayed that sex hormones might interact with HBV infection process and lead to a sex disparity. Wang et al. evaluated sex disparity of HBV infection by using HBV transgenic mice and cell culture system, and found ligand-stimulated androgen receptor could increase transcription of HBV RNAs through its transcription activation domain[30]. Wang et al. also found estrogen can repress transcription of HBV genes by up-regulating estrogen receptor-α, which interacts with and alters binding of hepatocyte nuclear factor-4α to the HBV enhancer I by using transgenic mice and HepG2 cells.[31]. There were more men than women in our CHB group than Clear group. To decrease bias of gender on effect estimates, we conducted the stratified analysis for sex. For SNP rs10485138 located in ASCC3 gene, a notable association was found in allele analysis for females. The proportions of the T and C allele were 31% and 69% respectively in CHB group, which were significantly different from those in Clear group (22% and 78% respectively). For SNP rs10485138, associations in genotype distributions for females and analysis for males and full sample were not observed. Hence, we only concluded that genetic variant rs10845858 in ASCC3 gene might slightly affect HBV persistence in Han Chinese females. The small sample for female HBV patients in this study might be the major reason for this weak association in female Chinese. More female samples and subjects from multiple areas are needed to verify the association in future. SNP rs10485138 was selected from a GWAS for HBV progression, which was performed by using DNA pools. Each pool was constructed by tens of participants. Specific information of each participant could not be obtained. So genotype frequencies of each SNP for males and females separately in the original GWAS study were not distinct. Genetic predisposition of SNP rs10485138 for other viruses or diseases was not found in PubMed database. So reasons why there is a difference between male and female for HBV infection need to be further studied.

Most HBV carriers are considered to be infected through maternal transmission in the neonatal period or infancy in high prevalent areas particularly in China [32–34]. The likelihood of developing CHB depends on age at the time of infection: 80–90% of infants infected during the first year of life and 30–50% of those aged under 5 years and less than 5% of healthy adults[5]. A relation about age and acute hepatitis B virus infection by McMnhon et al found that the risk of becoming a HBV carrier was inversing related to age of the patient at the time of infection[35]. In CHB group, minimum age was 16 and 79 patients were less than 35, while in Clear group, all subjects were 35 years or older to ensure spontaneously recovered from HBV infection. To decrease bias of age on effect estimates, a stratified analysis for age was performed. As a viral factor, hepatitis B e antigen (HBeAg) status was an important factor that associated with chronic HBV infection. A report about HBeAg and vertical transmission found that approximately 90% of HBeAg-seropositive mothers (with high viral load) transmit hepatitis B virus to their offspring compared with 10–20% of HBeAg-seronegative carrier mothers[36]. In this analysis, ninety-five CHB patients were HBeAg positive status. SNP rs10485138 had modest susceptibility with persistent HBV infection in females. We further calculated association between SNP rs10485138 and HBeAg status in CHB group. Significant association was not found. This phenomenon might be in accordance with viewpoint that genetic background and viral factor were two independent factors affecting persistent HBV infection.

HBV is a hepadnavirus, which has a strong preference for infecting liver cells. The main feature of the hepadnavirus replication cycle is the replication of the DNA genome by reverse transcription of an RNA intermediate. In the cycle, HBV genomes are repaired to form a covalently closed circular DNA (cccDNA), which is the template for viral messenger RNA (mRNA) transcription. HBV replication cycle is not directly cytotoxic to cells. After infection HBV, host immune responses to viral antigens are the principal determinants of hepatocellular injury. T-cell responses, especially the responses of cytotoxic T lymphocytes, play a central role in viral clearance. Reports about ASCC3 gene display that ASCC3 gene encodes a 3′-5′ DNA helicase[25], involved in DNA repair, cell proliferation[25] and functioned as a negative regulator of host defense response[37]. We suppose that ASCC3 gene might take part in HBV replication, clear or host immune responses to viral antigens. Further functional studies are required to establish the role of ASCC3 gene in pathogenesis of persistent HBV infection.

## Supporting Information

S1 Table Recruitment criteria for chronic hepatitis B (CHB) group and HBV patients who cleared their infection spontaneously (Clear) group. (DOCX)

S2 Table Taqman assay of eight SNPs (rs11866328, rs10845858, rs1041236, rs2013562, rs786100, rs12206945, rs10485138, rs6909880). (DOCX)

S3 Table Results of Hardy-Weinberg equilibrium for eight SNPs (rs11866328, rs10845858, rs1041236, rs2013562, rs786100, rs12206945, rs10485138, rs6909880). (DOCX)
