# Supplementary material for: Correlations between ASCC3 Gene Polymorphisms and Chronic Hepatitis B in a Chinese Han Population
Source: PLoS One. 2015 Nov 4;10(11):e0141861. doi: 10.1371/journal.pone.0141861 (PMC4633062; doi:10.1371/journal.pone.0141861)
Supplement: S1 Table — (DOCX) [file pone.0141861.s001.docx]

**S1 Table.** Recruitment criteria for chronic hepatitis B (CHB) group and HBV patients who cleared their infection spontaneously (Clear) group.

| CHB |
| --- |
| 1. HBsAg and anti-HBc positive for at least 6 months; |
| 2. Anti-HCV and HCV RNA negative; |
| 3. Anti-HDV and/or HDVAg negative; |
| 4.ALT and/or AST levels greater than 2 times upper limits of normal range for testing hospital before or current; |
| Clears |
| 1. Anti-HBs and anti-HBc positive; |
| 2. HBV-DNA negative, HDVAg negative and/or anti-HDV negative; |
| 3. Anti HCV and HCV RNA negative; |
| 4. ALT <40 IU/L and AST <45 IU/L at enrollment; and |
| 5. Age ≥ 35. |
