# Supplementary material for: Correlations between ASCC3 Gene Polymorphisms and Chronic Hepatitis B in a Chinese Han Population
Source: PLoS One. 2015 Nov 4;10(11):e0141861. doi: 10.1371/journal.pone.0141861 (PMC4633062; doi:10.1371/journal.pone.0141861)
Supplement: S2 Table — (DOCX) [file pone.0141861.s002.docx]

**S2 Table.** TaqMan assays of eight SNPs.

| SNP | Assay |
| --- | --- |
| [rs11866328](http://www.ncbi.nlm.nih.gov/SNP/snp_ref.cgi?rs=11866328) | C__11872152_10 |
| [rs10845858](http://www.ncbi.nlm.nih.gov/SNP/snp_ref.cgi?rs=10845858) | C__2680678_10 |
| [rs1041236](http://www.ncbi.nlm.nih.gov/SNP/snp_ref.cgi?rs=1041236) | C__2017276_20 |
| [rs2013562](http://www.ncbi.nlm.nih.gov/SNP/snp_ref.cgi?rs=2013562) | C__8857175_10 |
| [rs7861010](http://www.ncbi.nlm.nih.gov/SNP/snp_ref.cgi?rs=7861010) | C__29147493_20 |
| [rs12206945](http://www.ncbi.nlm.nih.gov/SNP/snp_ref.cgi?rs=12206945) | C__2460035_10 |
| [rs10485138](http://www.ncbi.nlm.nih.gov/SNP/snp_ref.cgi?rs=10485138) | C__29858049_20 |
| [rs6909880](http://www.ncbi.nlm.nih.gov/SNP/snp_ref.cgi?rs=6909880) | C__29435884_20 |
