# Supplementary material for: Correlations between ASCC3 Gene Polymorphisms and Chronic Hepatitis B in a Chinese Han Population
Source: PLoS One. 2015 Nov 4;10(11):e0141861. doi: 10.1371/journal.pone.0141861 (PMC4633062; doi:10.1371/journal.pone.0141861)
Supplement: S3 Table — (DOCX) [file pone.0141861.s003.docx]

**S3 Table.** Results of Hardy-Weinberg equilibrium test for eight SNPs.

| Polymorphisms | Allele | Clear (n=382) | CHB (n=493) |
| --- | --- | --- | --- |
| [rs11866328](http://www.ncbi.nlm.nih.gov/SNP/snp_ref.cgi?rs=11866328) | TT | 22 | 17 |
|  | TG | 123 | 159 |
|  | GG | 237 | 317 |
|  | T | 167 | 193 |
|  | G | 597 | 793 |
|  | Hardy‑Weinberg equilibrium | 0.262 | 0.589 |
| [rs10845858](http://www.ncbi.nlm.nih.gov/SNP/snp_ref.cgi?rs=10845858) | AA | 59 | 87 |
|  | AG | 205 | 254 |
|  | GG | 118 | 152 |
|  | A | 323 | 428 |
|  | G | 441 | 558 |
|  | Hardy‑Weinberg equilibrium | 0.052 | 0.28 |
| [rs1041236](http://www.ncbi.nlm.nih.gov/SNP/snp_ref.cgi?rs=1041236) | TT | 208 | 253 |
|  | TC | 154 | 198 |
|  | CC | 20 | 42 |
|  | T | 570 | 704 |
|  | C | 194 | 282 |
|  | Hardy‑Weinberg equilibrium | 0.211 | 0.712 |
| [rs2013562](http://www.ncbi.nlm.nih.gov/SNP/snp_ref.cgi?rs=2013562) | CC | 81 | 98 |
|  | CT | 187 | 246 |
|  | TT | 114 | 149 |
|  | C | 349 | 442 |
|  | T | 415 | 544 |
|  | Hardy‑Weinberg equilibrium | 0.791 | 0.846 |
| [rs7861010](http://www.ncbi.nlm.nih.gov/SNP/snp_ref.cgi?rs=7861010) | AA | 30 | 32 |
|  | AG | 148 | 203 |
|  | GG | 204 | 258 |
|  | A | 208 | 267 |
|  | G | 556 | 719 |
|  | Hardy‑Weinberg equilibrium | 0.663 | 0.344 |
| [rs12206945](http://www.ncbi.nlm.nih.gov/SNP/snp_ref.cgi?rs=12206945) | GG | 26 | 38 |
|  | GA | 144 | 185 |
|  | AA | 212 | 270 |
|  | G | 196 | 261 |
|  | A | 568 | 725 |
|  | Hardy‑Weinberg equilibrium | 0.818 | 0.424 |
| [rs10485138](http://www.ncbi.nlm.nih.gov/SNP/snp_ref.cgi?rs=10485138) | TT | 15 | 14 |
|  | TC | 120 | 155 |
|  | CC | 247 | 324 |
|  | T | 150 | 183 |
|  | C | 614 | 803 |
|  | Hardy‑Weinberg equilibrium | 0.929 | 0.374 |
| [rs6909880](http://www.ncbi.nlm.nih.gov/SNP/snp_ref.cgi?rs=6909880) | GG | 38 | 32 |
|  | GT | 153 | 186 |
|  | TT | 191 | 275 |
|  | G | 229 | 250 |
|  | T | 535 | 736 |
|  | Hardy‑Weinberg equilibrium | 0.37 | 0.942 |
